# A novel combined intelligent algorithm prediction model for the risk of the coal and gas outburst

**DOI:** 10.1038/s41598-023-43013-0

**Published:** 2023-09-25

**Authors:** Zhie Wang, Jingde Xu, Jun Ma, Zhuowen Cai

**Affiliations:** 1 School of Management Engineering, Capital University of Economics and Business Beijing, Beijing, 100083 China; 2 Institute of Higher Education, North China Institute of Science and Technology, Langfang, 065000 Hebei China; 3 School of Safety Science and Engineering, Anhui University of Science and Technology, Huainan, 232001 Anhui China

**Keywords:** Coal, Risk factors

## Abstract

The mechanism of coal and gas outburst disasters is perplexing, and the evaluation methods of outburst disasters based on various sensitive indicators often have some imprecision and fuzziness. With the concept of accurate and intelligent mining in coal mines proposed in China, selecting quantifiable parameters for machine learning risk prediction can avoid the deviation caused by human subjectivity, and improve the accuracy of coal and gas outburst prediction. Aiming at the shortcomings of the support vector machine (SVM) such as low noise resistance and being prone to be influenced by parameters easily, this research proposed a prediction method based on a grey wolf optimizer to optimize the support vector machine (GWO-SVM). To coordinate the global and local optimization ability of the GWO, Tent Chaotic Mapping and DLH strategies were introduced to improve the optimization ability of the GWO and reduce the local optimal probability. The improved prediction model IGWO-SVM was used to predict the coal and gas outburst. The results showed that this model has faster training speed and higher classification prediction accuracy than the SVM and GWO-SVM models, which the accuracy rate reaching 100%. Finally, to obtain the correlation between the parameters of the coal and gas outburst prediction parameters, the random forest algorithm was used for training, and the three parameters with the highest feature importance were selected to rebuild the data set for machine learning. The accuracy of the IGWO-SVM outburst prediction model based on Random Forest was still 100%. Therefore, even if some prediction parameters are missing, the outburst can still be effectively predicted by using the RF-IGWO-SVM model, which is beneficial for the model application and underground safety management.

## Introduction

**Figure 1:**
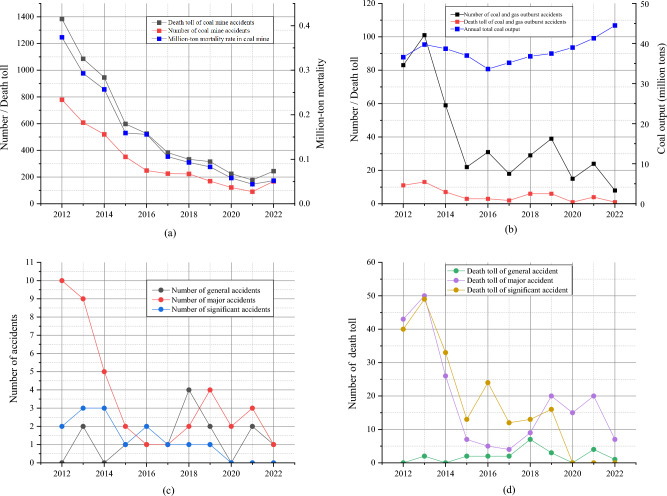
Statistical analysis of coal mine accidents and coal and gas outburst accidents in China from 2012 to 2022. (**a**) the data of Chinese coal mine accidents and million-ton mortality; (**b**) the data of gas outburst accidents and annual total coal output; (**c**) the variations in the number of kinds of coal and gas outburst accidents; (**d**) the variations in the death toll of kinds of coal and gas outburst accidents.

In 2022, the total output of coal mines in China is 4.45 billion tons, and coal consumption accounts for 56.2% of primary energy consumption^1^. In the future, coal will still be the most important basic energy in China, and it will play an indispensable role in economic and social development. According to the accident statistics published by the websites of the Ministry of Emergency Management of the People’s Republic of China annually, with the exploitation of coal resources, coal mine accidents can never be completely avoided, and the number of coal mine accidents in 2022 increased compared with that in 2021, which can be seen from Fig. 1a. During the decade from 2012 to 2022, 68 coal and gas outburst accidents occurred, resulting in 429 deaths as shown in Fig. 1b. In Fig. 1c and d, the number of coal and gas outburst accidents and deaths showed a downward trend as a whole, while fluctuating and rising in recent years, indicating that major accidents were effectively controlled as a whole. The coal and gas outburst is a dynamic disaster in which the gas-bearing coal in the mine suddenly moves rapidly from the coal seam to the mining space, accompanied by a large amount of gas emission, which seriously threatens the life and property safety of coal mine workers. China, Russia, the United States, Poland, Australia, and other major coal-mining countries have continuously invested abundant resources to actively solve the grievous disaster. China is one of the countries with the most severe dynamic disasters in the world.

At present, there are still about 840 high-gas mines and 719 coal and gas outburst mines in China. Besides, with the increasing mining depth, some low-gas mines have evolved into high-outburst mines, and gas burning and explosion accidents and gas overrun in the working face still occur from time to time^2^. Gas content is often used to evaluate outburst risk in coal mines, but due to the change in mining conditions, gas pressure, coefficient of coal firmness, and permeability should also be used to evaluate outburst risk in coal mines, and appropriate outburst threshold limits and controls should be determined^3^. While more and more coal mines are exploiting areas of high gas content and low permeability, the combination of these two factors often reduces the efficiency and effectiveness of gas drainage. It is difficult to reduce the gas content in the coal seam to below the outburst threshold. To control the risk, a large number of experts and scholars have explored the mechanism of outbursts and made great progress. Among them, the comprehensive action hypothesis of outburst is widely recognized, which reveals that outburst is the result of the joint action of the physical and mechanical properties of stress, gas, and coal^4^. Gas affects the effective stress of coal through the adsorption expansion effect and pore pressure. The adsorption expansion effect changes the contact between coal particles and affects the overall bearing of stress, while free gas in pores shares part of the external load^5^. The description of the mechanical behavior of coal-containing gas is mostly based on Terzaghi's effective stress theory, and the elastic–plastic model is used to explain the change, elastic deformation, and failure of the stress path. Experimental evidence suggests that the higher the gas pressure, the greater the expansion deformation, the lower the porosity, and the worse coal seam permeability^6^. It is assumed that under the comprehensive influence of stress, pore pressure, and temperature, the permeability of the coal seam in front of the working face may suddenly increase, causing coal and gas outburst disasters by studying the mechanical properties under various loading and unloading conditions^7^. Xue et al. and Wang et al. through the mechanical test of gas-bearing coal and coal-rock combination under triaxial conditions, the results show that the deformation of coal adsorbed gas has a significant impact on permeability^8,9^. Wang et al. suggested that gas desorption weakens the strength of coal, which is considered to be due to the reduced effective stress and the impact of the internal gas release on coal^10^. Sobczyk and Skoczylas studied the influence of adsorption and desorption processes on outbursts, discussed the danger of outburst of gas-bearing coal with different strength, then concluded that stress and mechanical properties of coal were the main factors determining outbursts strengths^11,12^. Ma and Xue quantitatively studied the energy intensity threshold of coal and gas outbursts under different gas pressures through laboratory tests^13,14^. Compared with gas in free pore gas energy, energy from desorption gas accounts for about 90% of outburst effective energy. Therefore, the laboratory experiments result show that coal and gas outburst is driven not only by gas but also by stress, and coal seam conditions.

The multi-factor theory provides a solid theoretical basis for the risk assessment and prediction of coal and gas outburst, and the research on the risk assessment of coal and gas outburst based on the comprehensive action hypothesis combined with various indicators is plentiful^15–19^. The mechanism of coal and gas outburst disasters is complex, and the evaluation methods of outburst disasters based on various sensitive parameters often have some inaccuracies and fuzziness. Many studies have been conducted based on prediction algorithms and models in coal mines^20–25^. With the concept of accurate and intelligent mining in coal mines put forward, researchers pay more and more attention to machine learning to predict coal and gas outbursts^26–29^. Such as, Particle Swarm Optimization (PSO) is used to optimize the prediction model of the back propagation (BP) algorithm^26^, random forest model is used to predict coal and gas outbursts^27^, and genetic algorithm is used to optimize the support vector machine model^28^. Given the lack of sample data, Zheng et al. used the data mining Multiple Imputation (MI) method to fill the missing data, and used the support vector machine (SVM) to predict coal and gas outburst^29^. The above models have significantly improved the prediction precision of coal and gas outburst, but there are still some improvements: for example, the optimization function of the GA algorithm needs to adjust more parameters, and the convergence speed of the GA needs to be improved, while the PSO algorithm has a fast convergence speed, but easily falls into local optimization, SVM, BP and other algorithms are weak at generalizing, which reduces the predicting power to some extent. The grey wolf optimization algorithm^30^ is a new type of swarm intelligence optimization algorithm. This algorithm optimizes the search by learning the leadership mode of the grey wolf population and the process of hunting prey. It has the characteristics of a simple principle, few parameter settings, and strong global search capability, but it has the drawbacks of strong development capability and weak exploration capability, and it is easy to fall into local optimization in the later stages. To solve this problem, the grey wolf optimizer algorithm can be improved by extracting features of the predictive parameters, using Tent Chaotic Mapping and DLH strategy^31^.

Given the aforementioned analysis, the mechanism and development process of coal and gas outbursts were investigated, and the outburst prediction parameters reasonably were determined. The data samples of Pansan Mine in Huainan Basin were tested, and the core parameters of the support vector machine were optimized by IGWO. The prediction accuracy of coal and gas outburst was compared between SVM and IGWO-SVM, which showed the IGWO-SVM model could accurately predict coal and gas outburst, and the effectiveness and superiority of the model were verified. Finally, the prediction parameters were extracted by Random Forest and trained by the models, the prediction results showed that the accuracy of the models was not reduced when three prediction parameters were selected. Therefore, Random Forest can effectively reduce the problems of diversified data types and large data volumes. The main contributions of this study are as follows:

1. According to the multi-factor outburst theory and energy conversion theory, the prediction parameters were reasonably determined, and the prediction parameters did not need complex or subjective data processing;
2. The grey wolf algorithm was improved and its effectiveness was verified;
3. Coal and gas outburst can be accurately predicted using the IGWO-SVM model;
4. The random forest algorithm was used to extract the features of the parameters, and the accuracy of the prediction models was not reduced by using the main parameters for training;
5. The superiority of the prediction model was verified by field and experimental data. It is helpful to accurately predict coal and gas outburst in coal mines for effectively preventing outburst disasters, formulating reasonable outburst prevention measures, improving the safety management level, and promoting the safety production capacity of coal mines.

## Foundations of the determination of prediction parameters

### Mechanism of the coal and gas outburst

#### Multi-factors theory of coal and gas outburst

**Figure 2:**
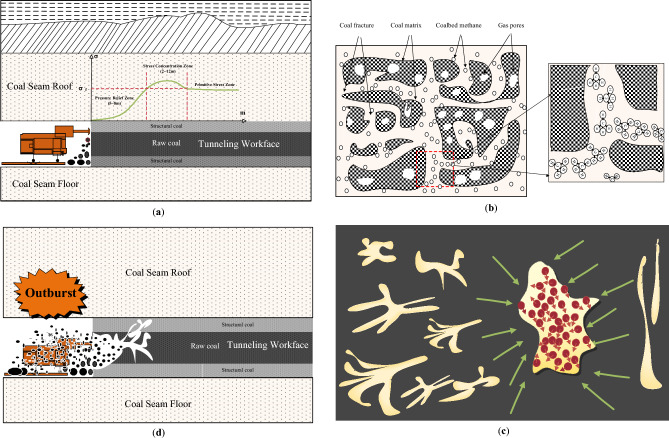
Mechanism of coal and gas outburst.

Multi-factor coal and gas theory is based on long-term human exploration of outburst and includes factors such as mine disturbance, effective stress, gas flow, and physical and mechanical properties of coal and rock. Currently, scientists have reached the same understanding that coal is damaged under the action of effective stress and the gas in coal can be quickly desorbed and the damaged coal can be discarded. Therefore, the outburst is the comprehensive effect of the dynamic distribution of geo-stress, gas in the coal seam, and the physical and mechanical properties of the coal seam itself. This has an impact on the gas extraction rate, desorption rate, gas pressure gradient, and mining efficiency. Coal and gas outburst is a mechanical failure process, and the outburst process can be divided into preparing, forming, developing, and ceasing processes as shown in Fig. 2. Li et al. stated that coal is subjected to tensile and shear failure under the action of in-situ stress and gas pressure^32^. In front of the working face, the coal seam is further crushed and destroyed under the action of the leading stress peak in Fig. 2a, which provides the necessary solid material base for the subsequent development of the outburst. The flow velocity of the gas in the pulverized coal is accelerated and the gas can accumulate, providing the necessary kinetic energy base for subsequent development. The dual-pore medium model of gas migration in coal is shown in Fig. 2b. Man-made disturbances such as blasting, tunneling, and pneumatic picking of coal cause a sharp change in the stress state of the coal and rock mass, and the pore pressure increases. This promotes the desorption of adsorbed gas and diffusion into the pores. As shown in Fig. 2c, when the coal seam in the outburst structure is suddenly exposed, a large amount of coal instantly rushes to the working face under the load of the gas, as shown in Fig. 2d. Therefore, the direct contribution of geological tectonic movement to outburst is to form a tectonic coal seam, create a high-stress environment conducive to outburst, and provide a geological structural environment conducive to gas preservation and outburst initiation. The conditions at the outburst site vary widely, but the outburst site must have a geological structural environment conducive to energy accumulation and sudden release.

The coal seam in the working face underwent a gradual increase from the initial original rock stress state to the peak stress state. With the occurrence of the unloading confining pressure, the peak stress exceeded the strength limit of the gas-bearing coal seam failure and seepage characteristics under stress perturbation, and the stress decreased to the residual stress state. In a gas-rich coal seam, the gas flow process in the coal is directly related to the stress state. In particular, the permeability is sensitive to the change in stress. As shown in Fig. 2a, the stress state of the coal seam went through a spatiotemporal evolution process, and the permeability of the coal seam changed correspondingly, and the gas flow showed a spatiotemporal characteristic as well. Coal permeabilities are influenced by gas pressure, adsorptive constants, porosity, and stress conditions, which are represented by changes in permeability gradients, adsorptive expansion characteristics of coal, and porosity loading. The effective stress formula is based on the Terzaghi principle and is defined as:

1 $${\sigma }_{ij}={{\sigma }_{ij}}^{\mathrm{^{\prime}}}-\alpha \cdot p$$

where: ${\sigma }_{ij}$ is effective stress; ${{\sigma }_{ij}}{\prime}$ is external stress; $p$ is pore pressure; $\alpha$ is effective stress coefficient.

The effective stress of the gas-bearing coal, taking into account the adsorption expansion of the coal, is expressed as:

2 $${\sigma }_{ij}={{\sigma }_{ij}}^{\mathrm{^{\prime}}}-{\sigma }_{s}-{\alpha }^{\mathrm{^{\prime}}}\cdot p$$

where: ${\sigma }_{s}$ is gas stress; ${\alpha }^{\mathrm{^{\prime}}}$ is the pore pressure coefficient.

Gas adsorption on coal reduces the surface energy of the coal, reducing the force between coal molecules and causing coal volume expansion, while desorption is the reverse process. The adsorption process affects the pore structure and the desorption process influences the pore structure. Macroscopically, coal exhibits adsorption expansion and desorption contraction effects. The expansion stress of coal adsorbing gas is given as:

3 $${\sigma }_{S}=\frac{2a\rho RT(1-2\mu )\mathrm{ln}(1+bp)}{3{V}_{m}}$$

where: $a$ and $b$ are Langmuir adsorption constants respectively; *R* is the universal constant of gas, 8.3143; *T* is the absolute temperature, K; $\mu$ is Poisson's ratio, $p$ is adsorption pressure; ${V}_{m}$ is the molar volume, 2.24 × 10^–3^ m^3^/mol.

Substituting (3) into formula (2) yields:

4 $${\sigma }_{ij}={{\sigma }_{ij}}^{\mathrm{^{\prime}}}-p\cdot \left[{\alpha }^{\mathrm{^{\prime}}}+\frac{2a\rho RT(1-2\mu )\mathrm{ln}(1+bp)}{3{V}_{m}p}\right]$$

With the redistribution of coal stress, the mechanical properties of coal and rock change, the increase or decrease in permeability will cause local variations. This will result in uneven distribution of the pressure gradient and uneven gas release during gas seepage. The permeability increases exponentially, the gas in the coal seam is rapidly released and a very high gas pressure gradient is created within the coal seam. This inevitably promotes coal and gas outburst.

#### Energy conversion theory of coal and gas outburst

**Figure 3:**
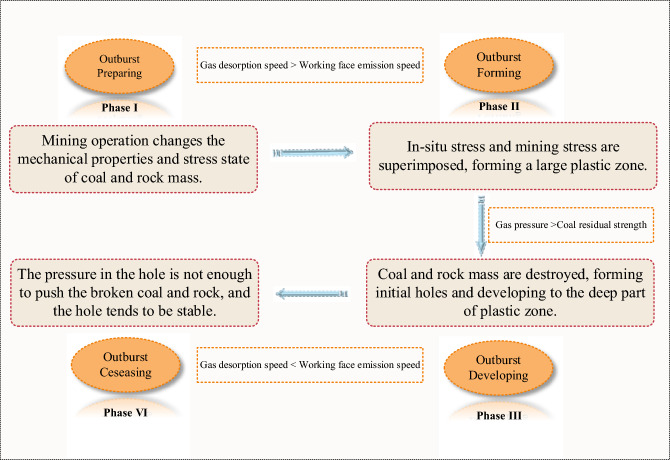
Description of the mechanical action process of coal and gas outburst.

Although the duration of an outburst is short, it still has its phase. From an energetic perspective, it generally has four stages as shown in Fig. 3. Phase I: The preparation stage of the outburst mainly completes the energy accumulation (elastic strain energy ${E}_{e}$, gas expansion energy ${E}_{p}$, and internal energy of coal $\Delta U$), and the direct cause of the outburst is related to the energy mutation. Phase II: The formation stage is the sudden instability, destruction, and ejection of the main structural coal seam, resulting in the exposure of the outburst coal seam. At this stage, it often manifests itself as sonic portents, such as the sound of machine guns, muffled thunder, and the sudden rise and fall of gas emissions. Phase III: The developing stage is the process from the exposure of the outburst coal to the end of the outburst. When it encounters a soft-hard coal junction or a new structure formed due to coal dust accumulation and hole clogging, the phenomenon of outburst deceleration, suspension, reignition, and initiation occurs. Phase VI: The ceasing stage is a point in time at the end of the outburst development. When the elastic strain energy, gas expansion energy, and internal energy of the coal and rock mass are less than the energy required for the outburst process (crushing work ${W}_{1}$, throwing work ${W}_{2}$, and energy loss of the gas flow field $\Delta E$), it will stop because the mechanical and energetic conditions are no longer favorable.

The outburst process is subject to the law of conservation of energy. During the occurrence and development of coal and gas outburst, the energy sources are elastic strain energy, gas expansion energy and coal internal energy, and crushing work, throwing work and gas flow field loss energy are the energy required for outburst. Therefore, the physical formula of the outburst process can be expressed as follows:

5 $${E}_{e}+{E}_{p}+\Delta U\ge {W}_{1}+{W}_{2}+\Delta E$$

where: ${E}_{e}$ is the elastic strain energy stored in the coal when stressed and is calculated as follows:

6 $${E}_{e}=\frac{1}{2E{\rho }_{c}}\left[{\sigma }_{1}^{2}+{\sigma }_{2}^{2}+{\sigma }_{3}^{2}-2\mu \left({\sigma }_{1}{\sigma }_{2}+{\sigma }_{2}{\sigma }_{3}+{\sigma }_{1}{\sigma }_{3}\right)\right]$$

where: ${\sigma }_{1}, {\sigma }_{2}, {\sigma }_{1}$ are stress in three directions, *E* is elastic modulus, MPa; ${\rho }_{c}$ is the density of coal, kg/m^3^.

${E}_{P}$ is the expansion energy of the gas, and the calculation formula is:

7 $${E}_{P}=\frac{{p}_{0}{V}_{0}}{\gamma -1}\left[{\left(\frac{{p}_{gas}}{{p}_{0}}\right)}^{\frac{\gamma -1}{\gamma }}-1\right]$$

where: ${p}_{gas}$ refers to the gas pressure under standard conditions, which is atmospheric pressure, 0.1 MPa; ${V}_{0}$ is the gas volume involved in the outburst process, including desorbed gas content and free gas content, m^3^/t; γ is a multinomial index. Judging from most outburst cases, the outburst process is close to the adiabatic process, and the approximate calculation of γ can be 1.25.

Coal internal energy is coal seam internal energy, and coal seam internal energy change can be calculated by formula (10) when an outburst occurs and coal seam temperature decreases:

8 $$\Delta U=\vartheta \cdot cm\Delta T$$

where: $\vartheta$ is the non-uniformity coefficient; $c$ is the specific heat capacity of coal, 0.79 kJ/(kg·K); $m$ is the quality of coal, kg/m^3^; $\Delta T$ is the falling temperature.

The formulae for calculating the crushing and throwing work per unit mass, based on the principle of increasing energy consumption per unit area, are as follows:

9 $${W}_{1}=\frac{3A\omega }{\rho }\sum_{i=1}^{n} \frac{{m}_{i}}{m}\cdot \left(\frac{1}{{d}_{i}}-\frac{1}{{d}_{0}}\right)$$

10 $${\mathrm{W}}_{2}=\frac{1}{2m}\sum_{i=1}^{n} {m}_{i}{v}_{i}^{2}$$

where: *A* is the energy consumed to increase the unit surface area is 505 J/m^2^; ${d}_{0}$ is the average particle size of the initial coal sample; ${d}_{i}$ is the average particle size of coal at a specific position; ω is the non-uniformity coefficient, 1.2 ~ 1.7.

The energy lost in the gas flow field is the kinetic energy of the gas, which corresponds to the energy dissipated after the gaseous and solid phases of the coal have separated. Theoretically, the formula is the following:

11 $$\Delta E=\frac{1}{2}{V}_{0}{\rho }^{\mathrm{^{\prime}}}{v}^{\mathrm{^{\prime}}2}$$

where: ${\rho }^{\mathrm{^{\prime}}}$ and ${v}^{\mathrm{^{\prime}}}$ is the density and velocity of gas flow when the gas phase and solid phase are separated.

Through the simulation test of coal and gas outburst in the laboratory, the calculation results of the energy in the outburst process are obtained. Of the total energy, 31% is used for the crushing work and 17% for the throwing work, and the loss of the gas flow field can reach 52%. A large amount of gas is released immediately. The energy change in the coal is used to promote the desorption behavior of the gas. The process of gas expansion and work transforms the internal energy of coal, so it can be considered that gas expansion energy ${E}_{P}$ and coal internal energy $\Delta U$ are gas-related energy, accounting for over 90% of total energy^13^. The gas-related energy is much greater than the elastic energy ${E}_{e}$ of coal, and the outburst is dominated by the energy associated with gas.

### Selection of predictive quantitative indicators

**Figure 4:**
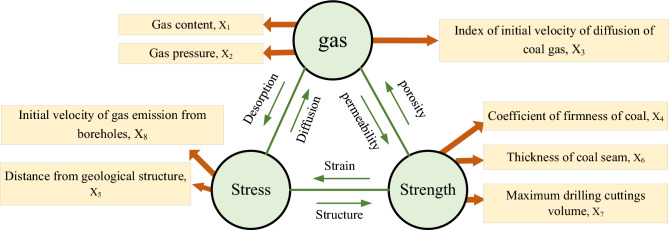
Schematic diagram of prediction parameters.

According to the mechanism of coal and gas outburst, eight quantitative parameters of outburst prediction can be summarized as shown in Fig. 4, including gas content, gas pressure, the initial velocity of gas emission from boreholes, index of the initial velocity of diffusion of coal gas, coefficient of firmness of coal, the thickness of the coal seam, maximum drilling cuttings volume, and distance from the geological structure. The relationship of each parameter to coal and gas outburst is explained. From Eq. (4), it is clear that when the coal seam is exposed or the coal seam in the working face is exposed, the gas pressure is reduced and the gas in the coal seam is desorbed and liberated, the expansion stress and pore pressure are reduced and the coal seam contracts, which also causes the effective stress to varying significantly. As the porosity of coal is low and nanoscale pores are the main ones, the influence of gas on coal mechanical properties is mainly the combined effect of expansion stress and pore pressure caused by micro-surface adsorption of a large amount of gas. Therefore, the influence of effective stress on the micropore structure of coal is an important factor in causing coal destruction. The magnitude and variation of the initial gas emission velocity and the initial gas emission velocity can characterize the internal stress variation of the coal seam. Gas pressure and gas content are important parameters affecting the internal energy and expansion energy of the gas. The coefficient of firmness of coal, the thickness of the coal seam, and maximum drilling cuttings volume can describe the internal energy of coal seam, while the distance from the geological structure is a parameter that characterizes the stress concentration area. Therefore, selecting a reasonable set of eight indicators related to prominence is important for subsequent machine learning.

## Theoretical foundations of the algorithms

This section may be divided by subheadings. It should provide a concise and precise description of the experimental results, their interpretation, as well as the experimental conclusions that can be drawn.

### Support vector machine

Support Vector Machine (SVM) was proposed by Vapnik in 1995. It is based on statistical learning to solve the problems of small sample size, nonlinearity and high dimensional identification, and is an optimization algorithm of convex quadratic programming^33^. Constructing an optimal hyperplane with maximum interval to achieve optimal classification between data is the core idea of SVM^25^. For example, a known training set, $Z\left\{\left({x}_{1},{y}_{2}\right),\dots ,\left({x}_{i},{y}_{i}\right)\right\}\in (X\times Y)l{x}_{i}\in x={R}_{n},{y}_{i}\in y=\{-\mathrm{1,1}\},i=\mathrm{1,2},\dots ,lZ$. Constructing a hyperplane to best divide the training set into the maximum interval. The hyperplane is called the prediction hyperplane and is expressed as follows:

12 $$y=\omega \cdot\Phi (x)+b$$

where: $y$ is the prediction function; $\omega$ is the weight; $b$ is biased.

However, some data cannot be hyperplane-predicted and the linearly separable SVM model fails due to the uneven distribution of the sample data sets. To solve the above problems, relaxation variables ${\xi }_{i}$ are introduced as boundary conditions to reflect the situation that some sample points are misclassified. At the same time, to avoid a too strong influence on the classification results, a penalty factor C is introduced to compensate for this influence. ${\xi }_{i}\sum_{i=1}^{n} {\xi }_{i}$ is defined as the misclassified part of the data set, and the linear constraint equation and constraint conditions are:

13 $$\begin{aligned} & \underset{w,b,{\xi }_{i}}{min} \frac{1}{2}\parallel \omega {\parallel }^{2}+C\sum_{i=1}^{n} {\xi }_{i} \\ & \text{s.t }{\mathrm{y}}_{\mathrm{i}}-{\upomega }^{\mathrm{T}}\cdot {\mathrm{x}}_{\mathrm{i}}-b\le {\upxi }_{\mathrm{i}} ({\upxi }_{\mathrm{i}}\ge 0,\mathrm{i}=\mathrm{1,2},\cdots \mathrm{m}) \end{aligned}$$

The Lagrange function is introduced to transform the equation into its dual form, and the solution of formula (12) can be obtained as follows:

14 $$f(x)=C\sum_{i=1}^{n} {\xi }_{i}K\left(x,{x}_{i}\right)+b$$

where: $K(x, {x}_{i})$ is the kernel function; $K(x, {x}_{i})=\mathrm{exp}\left(-g|x-{x}_{i}{|}^{2}\right)$, the Gaussian radial basis function with a wider application range is selected as the kernel function. The parameter g of kernel function determines the influence degree of kernel function on the support vector machine.

Therefore, the penalty factor C and the parameter g of the kernel function are the main parameters that affect the prediction performance of the SVM model.

### Grey wolf optimizer

Grey Wolf Optimizer (GWO) is inspired by the grey wolf. It stimulates the leadership and hunting mechanism of grey wolves in the wild. Among them, $\alpha , \beta ,\delta ,\omega$ represent the leader wolf, the auxiliary wolf, and the subordinate wolves of the third and fourth classes respectively. In addition, the three main steps of the hunt are realized: finding the prey, surrounding the prey, and attacking the prey.

#### Finding the prey

In the process of hunting, the distance between the grey wolf and the prey is expressed as:

15 $$D=\left|C\cdot {X}_{p}(t)-X(t)\right|$$

16 $$X\left(t+1\right)={X}_{p}\left(t\right)-A\cdot D$$

Formula (15) represents the distance between the individual and the prey, and formula (15) is the position updating formula of the grey wolf. Where: t is the current iterative algebra; A and C are coefficient vectors; ${X}_{p}(t)$ and $X(t)$ are the position vectors of the prey and the grey wolf respectively. The calculation formulas for A and C are as follows:

17 $$A=2a\cdot {r}_{1}-a$$

18 $$C=2\cdot {r}_{2}$$

where: $a$ is the convergence factor, which linearly decreases from 2 to 0 with the number of iterations; ${r}_{1}$ and ${r}_{2}$ are a random vector, taking a random number between [0,1].

#### Surrounding the prey

The grey wolf can identify the position of the prey and surround them. When the grey wolf identifies the position of prey, it can guide the wolves to surround the prey under the guidance wolf. The mathematical model of grey wolf tracking prey position is described as follows:

19 $$\left\{\begin{array}{c}{D}_{\alpha }=\left|{C}_{1}\cdot {X}_{\alpha }(t)-X(t)\right|\\ {D}_{\beta }=\left|{C}_{2}\cdot {X}_{\beta }(t)-X(t)\right|\\ {D}_{\delta }=\left|{C}_{3}\cdot {X}_{\delta }(t)-X(t)\right|\end{array}\right.$$

20 $$\left\{\begin{array}{c}{X}_{1}(t)={X}_{\alpha }(t)-{A}_{1}\cdot {D}_{\alpha }(t)\\ {X}_{2}(t)={X}_{\beta }(t)-{A}_{2}\cdot {D}_{\beta }(t)\\ {X}_{3}(t)={X}_{\delta }(t)-{A}_{3}\cdot {D}_{\delta }(t)\\ X(t+1)=\frac{{X}_{1}(t)+{X}_{2}(t)+{X}_{3}(t)}{3}\end{array}\right.$$

where: ${D}_{\alpha }$,${ D}_{\beta }$, and ${D}_{\delta }$ are the distance between $\alpha$, $\beta$ and $\omega$ with other wolves respectively; and are the optimal solution positions of three wolves respectively; ${X}_{\alpha }(t)$, ${X}_{\beta }(t)$, and ${X}_{\delta }(t)$ are the optimal solution positions of three wolves respectively.; ${A}_{1}$,${A}_{2}$, ${A}_{3}$, ${C}_{1}$, ${C}_{2}$, and ${C}_{3}$ are random vectors, ${X}_{1}(t)$, ${X}_{2}(t)$, and ${X}_{3}(t)$ are the moving direction and step size of grey wolves, $X(t+1)$ is the average distance between wolves.

#### Attacking the prey

**Figure 5:**
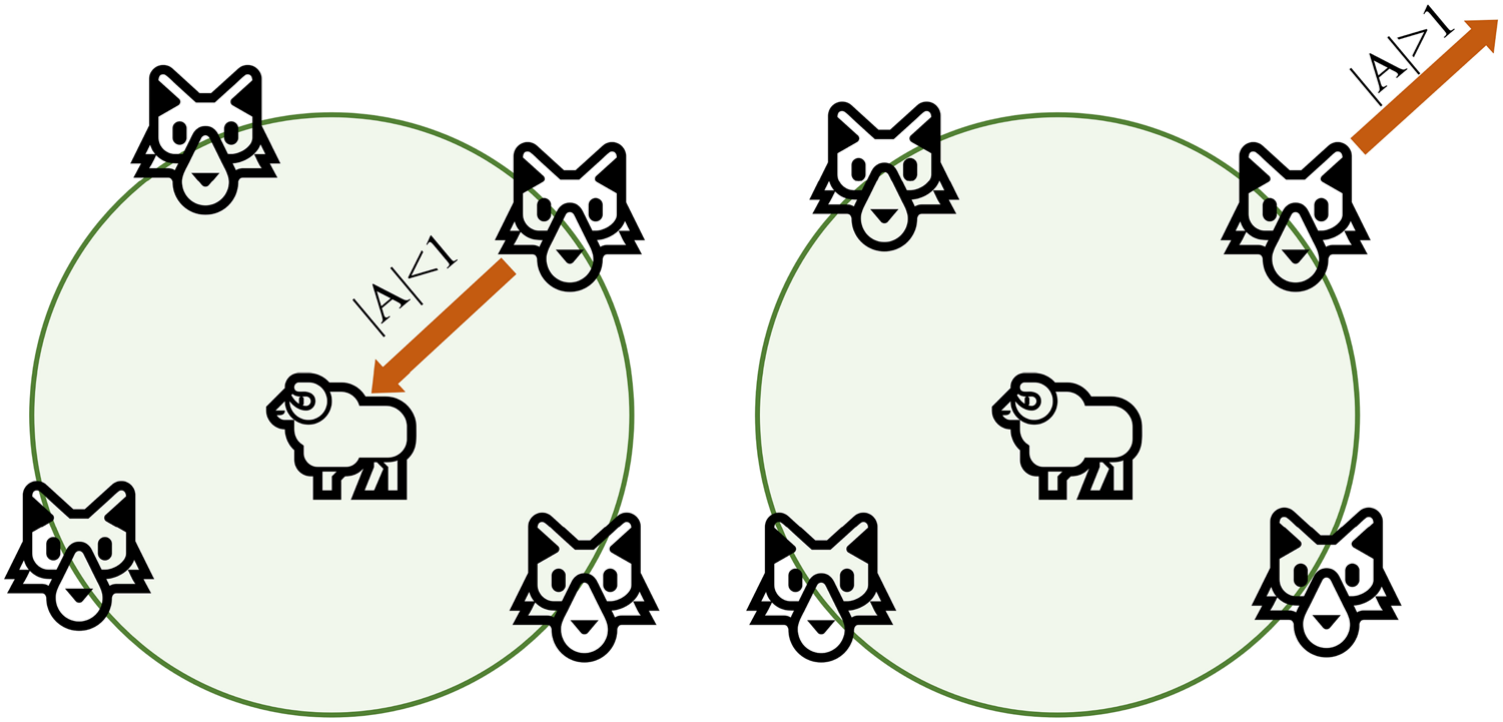
Schematic hunting diagram of the GWO algorithm.

When the prey stops moving, the grey wolf completes the hunt by attacking. For the simulation of approaching prey, the value is gradually reduced so that the fluctuation range is also reduced. In other words, as the value is linearly reduced from 2 to 0, the corresponding value also changes in the interval [− a, a]. As shown in Fig. 5, the next position of the grey wolf can be anywhere between the current position and the prey position if the value of A is in the interval. If |A| < 1, the grey wolf will attack its prey (falling into the local optimum). If |A| > 1, the grey wolf separates from the prey in the hope of finding more suitable prey (global optimum).

### Improved GWO

In GWO, *α*, *β*, and *δ* guide* ω* wolves into the search space to find the optimal solution domain, which would cause the GWO algorithm to converge slowly. Furthermore, since the convergence factor A decreases linearly with increasing iteration times, too small a value will gradually move the wolves away from the optimal solution position, leading to local optimization of the algorithm. To overcome the above problems, the tent chaotic map and DLH strategy are introduced.

Chaotic mapping produces a chaotic sequence instead of a pseudorandom number generator. In the GWO algorithm, whether the initial population is uniformly distributed has a great influence on the performance of the GWO algorithm. In the case of a uniformly distributed population, after many iterations, it can achieve a larger search area and faster convergence speed than random distribution. The expression of tent chaotic mapping is as follows:

21 $${x}_{k+1}=\left\{\begin{array}{ll}\frac{{x}_{k}}{0.6} & \quad {x}_{k}<0.6\\ \frac{5}{2}\left(1-{x}_{k}\right)& \quad {x}_{k}\ge 0.6\end{array}\right.$$

where: ${x}_{k}$ and ${x}_{k+1}$ are the chaotic sequences generated by the chaotic mapping, k = 0, 1, 2, … n.

The dimension of the learning-based hunting strategy (DLH) is to allow the wolves to learn from the wolves around them as they hunt.

#### Initialization stage

In this phase, wolves with a total number of n are randomly distributed in a given search area [${l}_{j}$, ${u}_{j}$] according to Eq. (9), where $D$ is the dimension of the problem, and the wolves form a matrix with $N$ rows and $D$ columns, which ${X}_{i}(t)$ is computed using the fitness function.

22 $${X}_{ij}={l}_{j}+{\mathrm{rand}}_{j}[\mathrm{0,1}]\times \left({u}_{j}-{l}_{j}\right),i\in [1,N],j\in [1,D]$$

#### Movement stage

Each wolf learns the strategies of its neighbors. It becomes another candidate for a new position. In GWO, α, β, and δ are the three best wolves. With the linear decrease of the coefficient and the positions of X_α_, X_β_ and X_δ_, the position of the surrounding prey is determined. Finally, the first candidate who moves to the new position of the grey wolf ${X}_{i}(t)$ is named ${X}_{i-GWO}(t+1)$. DLH strategy generates another candidate position ${X}_{i-DLH,d}(t+1)$ for wolves, and the calculation formula is as follows:

23 $${X}_{i-DLH,d}(t+1)={X}_{i,d}(t)+rand\times \left({X}_{n,d}(t)-{X}_{r,d}(t)\right)$$

where: ${X}_{n,d}(t)$ is a wolf nearby; ${X}_{r,d}(t)$ is a random wolf in the population.

The distance calculation formula is as follows:

24 $${M}_{i}(t)=\left\{{X}_{j}(t)\mid {D}_{i}\left({X}_{i}(t),{X}_{j}(t)\right)\le {R}_{i}(t),{X}_{j}(t)\in I\right\}$$

25 $${R}_{i}(t)={||X}_{i}(t)-{X}_{i-GWO}(t+1)||$$

where: ${R}_{i}(t)$ is the Euclidean distance between ${X}_{i}\left(t\right)$ and ${X}_{i-GWO}(t+1)$; ${M}_{i}(t)$ is the distance of ${R}_{i}(t)$ of wolves adjacent to ${X}_{i}(t)$; ${D}_{i}$ is the Euclidean distance between ${X}_{i}(t)$ and ${X}_{j}(t)$.

#### Selection and update stage

In this stage, the better candidate is selected by comparing the fitness values of the sum of the two candidates.

26 $${X}_{i}(t+1)=\left\{\begin{array}{cc}{X}_{i-GWO}(t+1),& \text{ if }f\left({X}_{i-GWO}\right)<f\left({X}_{i-DLH}\right)\\ {X}_{i-DLH}(t+1)& \text{ otherwise}\end{array}\right.$$

To update the new position ${X}_{i}(t+1)$, if the selected candidate's fitness is less than ${X}_{i}(t)$, it is updated with the selected candidate's position. Otherwise, it remains unchanged. Finally, after the process has been carried out on all the individuals, the number of iterations is increased by 1 until the iterative search has reached the pre-defined number of iterations.

### Random forest

**Figure 6:**
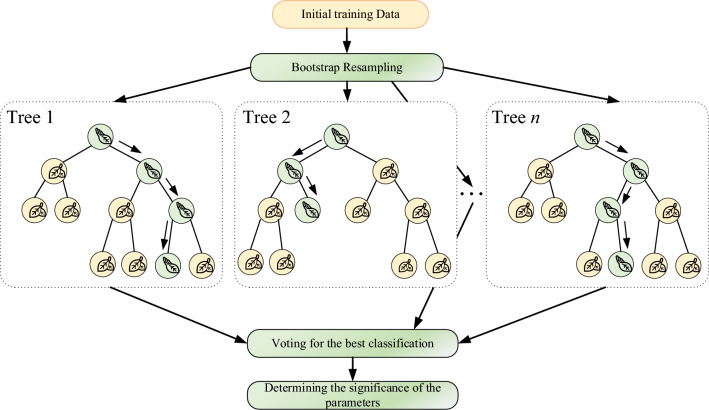
Schematic diagram of the Random Forest algorithm.

Random Forest is a commonly used method in machine learning for classifying data. Its model uses ensemble learning theory and the classification model uses a decision tree algorithm. Currently, an important problem in machine learning is overfitting. Random forest is an ensemble learning algorithm that belongs to the bagging type. By combining several weak classifiers, the final result is voted or averaged. This gives the result of the whole model a high accuracy and generalization performance. It can achieve good results mainly due to the "randomness" and "forest", the former making it anti-fitting and the latter making it more accurate. The specific steps of the random forest algorithm are illustrated in Fig. 6.

## Prediction model of the coal and outburst building

### Highlight the forecast parameter set

To construct a vector ***X* = *(X***_***1***_***, X***_***2***_***, X***_***3***_***, X***_***4***_***, X***_***5***_***, X***_***6***_***, X***_***7***_***, X***_***8***_***)***, select eight forecast indicators. The elements in the vector represent: ***X***_***1***_ is the gas pressure, ***X***_***2***_ is the gas content,*** X***_***3***_ is the index of the initial velocity of diffusion of coal gas, ***X***_***4***_ is the coefficient of firmness of coal, ***X***_***5***_ is the distance from geological structure, ***X***_***6***_ is the thickness of coal seams, ***X***_***7***_ is the maximum drilling cuttings volume, and ***X***_***8***_ is the initial velocity of gas emission from boreholes. Among ***X* = *(X***_***1***_***, X***_***2***_***, X***_***3***_***, X***_***4***_***, X***_***5***_***, X***_***6***_***, X***_***7***_***, X***_***8***_***)***, ***X***_***1***_ is the gas pressure, ***X***_***2***_ is the gas content,*** X***_***3***_ is the index of the initial velocity of diffusion of coal gas, and ***X***_***8***_ is the initial velocity of gas emission from boreholes are variates; ***X***_***4***_ is the coefficient of firmness of coal, ***X***_***5***_ is the distance from geological structure, ***X***_***6***_ is the thickness of coal seams, and ***X***_***7***_ is the maximum drilling cuttings volume are constant in a specific period. The data used in this prediction is from the measured data of the 8#, 11#, and 13# coal seams in the Pansan Mining Area of Huainan Basin, China. In addition, each group of data is divided into outburst danger areas according to the “Prediction Conclusion of Outburst Danger in Outburst Coal Seam Areas in Pansan Mining Area”, where 1 represents an area with outburst risk area and 0 represents an area without outburst danger. The data sets are divided into two categories to form an 84 × 9 data matrix. Each data set represents a different underground site as shown in Table 1, including 8 types of predictive index values measured at the site and the blast hazard in the area where the site is located。

**Table 1:** Normalized partial data set.

*X*_*1*_	*X*_*2*_	*X*_*3*_	*X*_*4*_	*X*_*5*_	*X*_*6*_	*X*_*7*_	*X*_*8*_	Risk
0.18	0.41	0.02	0.68	0.00	0.69	0.93	0.24	1.00
0.16	0.39	0.39	0.03	0.98	0.59	0.44	0.07	0.00
0.30	0.57	0.12	0.59	0.00	0.16	1.00	0.12	1.00
0.10	0.42	0.43	0.61	0.00	0.25	0.52	0.22	0.00
0.28	0.56	0.15	0.28	0.00	0.72	0.56	0.48	1.00
0.10	0.44	0.30	0.04	0.08	0.50	0.63	0.43	0.00
0.27	0.62	0.44	0.45	0.00	0.06	0.96	0.09	1.00
0.16	0.43	0.43	0.39	0.00	0.78	0.56	0.31	0.00
0.01	0.07	0.47	0.18	0.07	0.16	0.44	0.12	0.00
0.48	0.85	0.49	0.84	0.00	0.84	0.56	0.28	1.00
0.13	0.38	0.15	0.32	0.00	0.72	0.81	0.44	0.00
0.16	0.43	0.47	0.72	0.03	0.09	0.89	0.12	0.00
0.00	0.10	0.34	1.00	0.33	0.19	0.44	0.10	0.00
0.56	0.82	0.47	0.51	0.00	0.13	0.78	0.10	1.00
0.05	0.16	0.63	0.89	0.30	0.09	0.41	0.00	0.00
0.65	0.88	0.57	0.66	0.00	0.66	1.00	0.23	1.00
0.19	0.49	0.03	0.70	0.82	0.63	0.48	0.23	1.00
0.77	0.84	0.43	0.63	0.00	0.69	0.96	0.62	1.00
0.88	0.94	0.10	0.64	0.00	0.63	0.59	0.62	1.00
0.10	0.43	0.40	1.00	0.66	0.16	0.89	0.00	0.00

### Prediction model building

**Figure 7:**
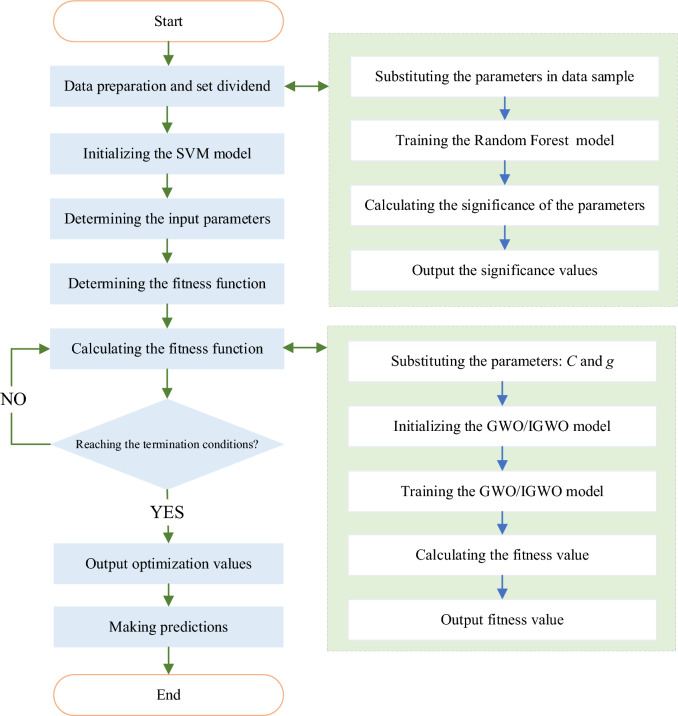
Diagram of processes of the prediction models.

The prediction model is an improved Grey Wolf optimizer coupled with SVM and Random Forest dimensionality reduction, as shown in Fig. 7. The basic process of prediction is as follows:

1. Standardize the raw data, and divide the training set and test sets. Preprocessing according to the collected sample dataset, using 8 kinds of prediction characteristic parameters to consist model, normalizing the size of all data except the classification result to [0, 1], then selecting 63 data groups from the dataset as training set for training, and the remaining 21 data groups as testing set to verify the prediction result;
2. The SVM is initialized, the optimal ***C*** and ***g*** for the test set are found out by the training set, and the prediction result of the SVM model is output; Based on the mathematical modeling software of MATLAB, the mathematical model of SVM is constructed by referring to the related functions of ***svmtrain*** and ***svmpredict*** in the ***libsvm*** toolbox, and the classified training set data is input into the model for training. The optimal penalty parameter ***C*** and kernel function parameter ***g*** obtained from the training set are substituted into the support vector machine model, and the test sample set is input to analyze the prediction performance of the trained support vector machine model.
3. Use the full sample data set constructed in Step 1, use GWO to find the optimal c and used for SVM prediction then output the pre-judgment results of the GWO-SVM model; Again, the full sample data set constructed in Step 1 is used, and DLH strategy is added to improve the grey wolf optimization algorithm to optimize the parameters of penalty parameter ***C*** and kernel function parameter ***g***, and the ability of IGWO-SVM model for sample classification of test set is verified.
4. Random forest algorithm is applied to obtain the feature importance of eight prediction indicators, and the sample dataset is reconstructed by screening indicators to make the results of multiple models have high accuracy and generalization performance. The best ***C*** and ***g*** are found by using IGWO. The prediction results verify the superiority of the RF-SVM-IGWO model.

## Results and discussions

### SVM

**Figure 8:**
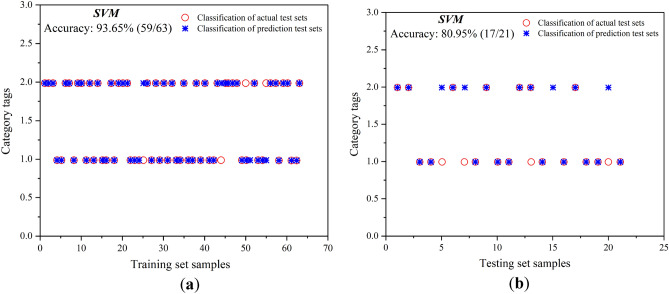
Predictive results of the SVM model. (**a**) Predictive results of the training set samples; (**b**) predictive results of the test set samples.

The sample set is divided according to a 3:1 ratio and 63 groups of data are randomly selected as the training set and 21 groups of data are randomly selected as the test set. The data is normalized to eliminate the influence of different dimensional indices on the model. Initialize the SVM model. Since the values of the penalty factor*** C*** and the kernel function ***g*** of the SVM model without parameter optimization are uncertain, an ***rng('default')*** function is introduced to set a random value for ***C*** and ***g*** as the parameters of the SVM for training and analysis. The distribution coordinates of the prediction results obtained by SVM are shown in Fig. 8.

From the prediction results of the SVM model without parameter optimization, it can be seen that the prediction accuracy of the training set is 93.65% and the accuracy of the test set is only 80.95%. The prediction of the test sets in groups 5, 7, 13, and 20 is wrong. The findings show that after repeated training of the SVM, a good fitting effect is achieved on the training set data, but the accuracy of the test set is average, indicating that the non-optimized SVM model does not have sufficient predictive ability for unknown data and still needs to be optimized. At the same time, it can be seen that the model performs well on the training set, but poorly on the test set. The results are quite different, indicating that the model's prediction results have been over-fitted to some extent. Therefore, to improve the predictive ability of the model for unknown data, the GWO grey wolf optimization algorithm is introduced to optimize the ***C*** and ***g*** parameters and improve the model performance.

### GWO-SVM

**Figure 9:**
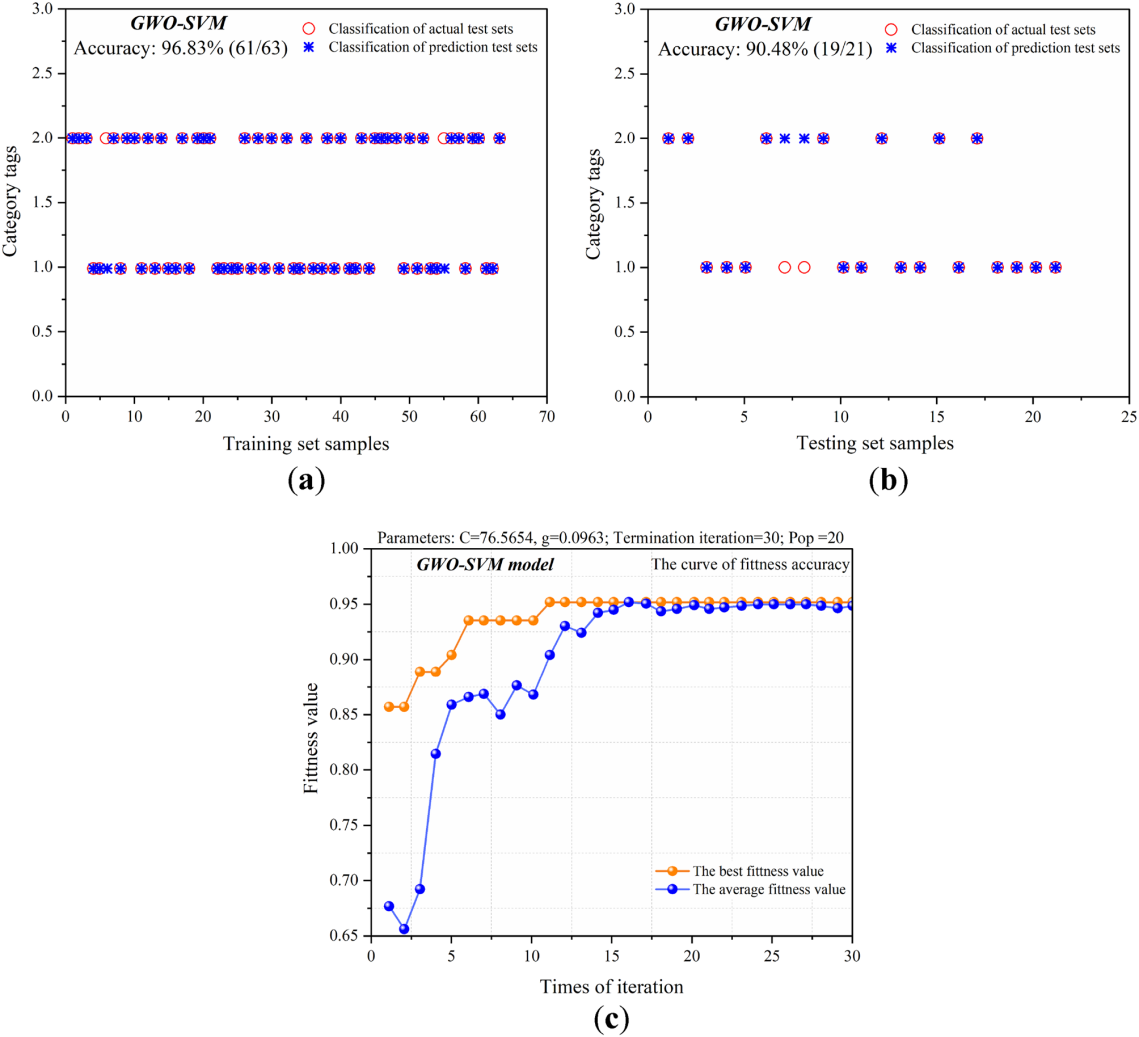
Predictive results of the GWO-SVM model. (**a**) Predictive results of the training set samples; (**b**) predictive results of the test set samples; (**c**) fitness curve of the GWO-SVM model.

The original datasets of the training and test sets are left unchanged, with the number of grey wolf populations set to 20, the maximum number of iterations set to 30, and the range of variation of the parameters ***C*** and ***g*** set to [0.1, 100]. Following the specific steps of the GWO, the corresponding mathematical model is constructed in MATLAB, and the predictive results from the operation are shown in Fig. 9.

From Fig. 9, the prediction accuracy of the optimized SVM model in the training set reached 96.83%. The prediction accuracy of the testing set reached 90.48%. The data of the seventh and eighth groups were wrong. It can be seen from the population fitness curve that in the process of population evolution iteration in Fig. 9c, the population fitness gradually increased from generation 0 to generation 7, and fluctuated after generation 8. The fitness decreased and then increased. The final average fitness value is stable between 90 and 95%, and the best fitness value is 95.24%.

Compared with the prediction results of the SVM model, it can be seen that the GWO-optimized SVM algorithm has achieved the best detection results in both the training set and the test set, and the accuracy of the test set has been significantly improved for the SVM model without parameter optimization, indicating that the prediction performance of the SVM model has been improved after the GWO algorithm optimization.

### IGWO-SVM

**Figure 10:**
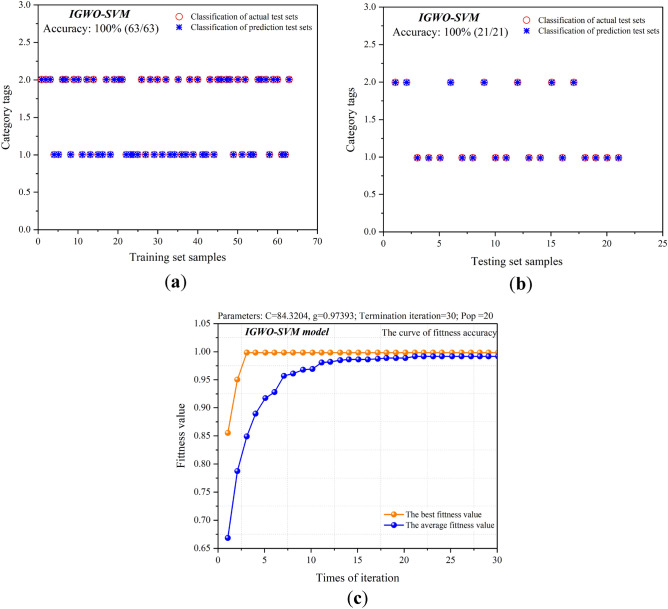
Predictive results of the IGWO-SVM model. (**a**) Predictive results of the training set samples; (**b**) predictive results of the test set samples; (**c**) fitness curve of the IGWO-SVM model.

The GWO-SVM algorithm has some overfitting due to the large difference between the results of the training and test sets. Moreover, without feature dimensionality reduction and algorithm optimization, the highest accuracy of the test set obtained by the two models is only 90.48%, so there is still room for improvement in the ability to predict unknown data. Tent chaotic mapping and DLH strategy are introduced to improve the GWO algorithm. Through the improved GWO model, the optimal ***C*** and ***g*** parameters for SVM for outbreak prediction are found, and the results are shown in Fig. 10.

From the results, it can be seen that IGWO has a good effect in optimizing the SVM model, and the accuracy of the training set is 100%, and the accuracy of the test set is 100%, which not only improves the accuracy of the test set, but also narrows the gap between the prediction accuracy of the test set and the training set, solves the over-fitting problem, and improves the accuracy of the test set. From the fitness curve, the fitness of the population gradually increased from Generation 0 to Generation 10. There was no fluctuation in that the fitness suddenly decreased and then increased until the evolution to generation 30. The final average fitness was stable between 0.95 and 1, and the best fitness was 1. Therefore, the effectiveness of the improved GWO by the Tent Chaotic Mapping and the DLH strategy is verified.

### Random forest for the dimension reduction

To better determine the influence of each parameter on the data, it is necessary to reduce the dimensionality of the data, eliminate irrelevant redundant features, determine the optimal feature set, and more accurately guide the classification problem. The number of random forest parameter trees is set to 500 and the number of leaves is set to 4. The random forest algorithm is used to reduce the dimension of the data. The results of the feature importance values calculated by random forest are shown in Table 2.

**Table 2:** The results of the feature important value.

No.	Parameters	Feature importance value
X_1_	Gas content, m^3^/t	0.7537
X_2_	Gas pressure, MPa	1.0803
X_3_	Index of the initial velocity of diffusion of coal gas, mmHg	0.1454
X_4_	Coefficient of firmness of coal	0.3668
X_5_	Distance from geological structure, m	0.0207
X_6_	The thickness of coal seams; m	0.2474
X_7_	Maximum drilling cuttings volume, kg/m	0.1865
X_8_	The initial velocity of gas emission from boreholes, L/min	0.0801

The importance values of the gas pressure (X_2_), gas content (X_1_), and coefficient of firmness of coal (X_4_) are greater than 0.3 in rank 1–3. The importance values of gas content (X_1_) and gas pressure (X_2_) of the first and second features are higher than those of other features, and the importance values are all greater than 0.6, which represent two main parameters that are focused on and detected in the daily mining activities of coal mines, and have a certain guiding role in the prevention and control of coal and gas outburst. The smaller the coefficient of firmness of coal, the lower the hardness of the coal seam, the higher the porosity, and the more gas will be adsorbed in the coal seam, increasing the risk of coal and gas outburst. Therefore, as a predictive index for coal and gas outburst areas, the characteristics ranked 1–3 are closely related to the presence of the outburst risk.

**Figure 11:**
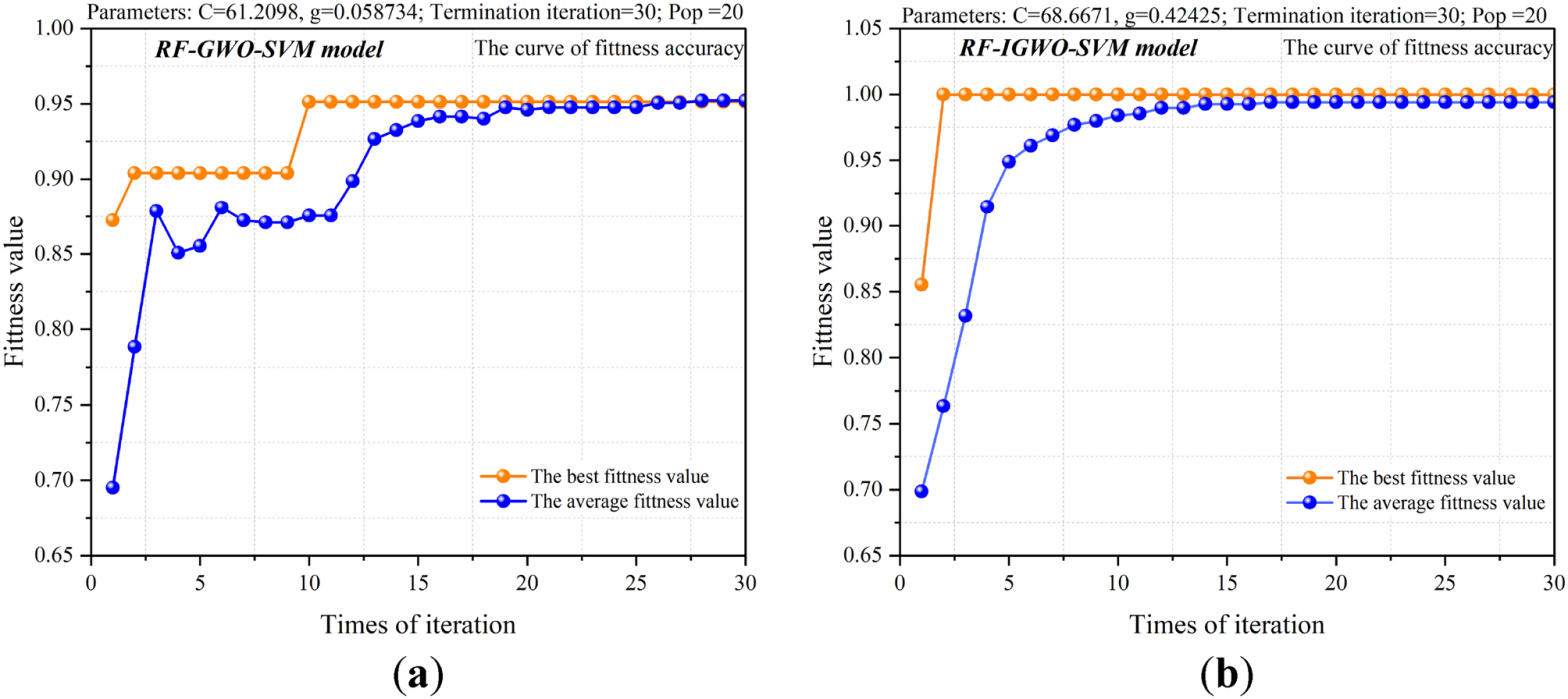
Fitness curve of multiple models. (**a**) Fitness curve of the RF-GWO-SVM model; (**b**) fitness curve of the RF- IGWO-SVM model.

**Figure 12:**
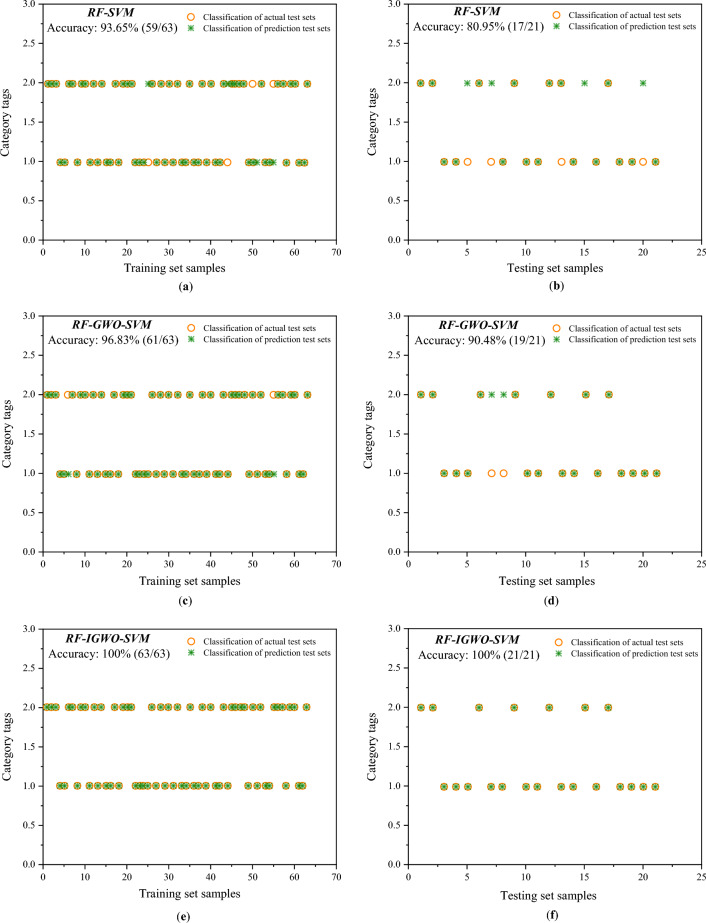
Diagram of results of the prediction models with 3 predictive parameters.

Taking the top three characteristics (gas content, gas pressure, and coefficient of firmness of coal) as the results of random forest dimension reduction, the data set is reconstructed. Then the SVM, GWO-SVM, and IGWO-SVM models are used to train and test the reconstructed data set and the fitness curve and predictive results are obtained as shown in Figs. 11 and 12. The random forest algorithm can effectively improve fitness and quickly reach equilibrium, as shown in Fig. 11. It can be seen that the accuracy of the model has not decreased, and the prediction accuracy of the three main indicators is consistent with that of the eight indicators when comparing the prediction results of the model in Figs. 8, 9, 10, and 12.

Compared to the results of parameter training without random forest as shown in Table 3, it can be seen that the training speed and accuracy of the model have not changed. Note that the training accuracy of the RF-IGWO-SVM model is still 100% compared with the IGWO-SVM model, indicating that when some data are difficult to measure in the practical application of the model and the data are lacking, one can still get good predictive results by using only three characteristics of gas pressure, gas content and coal strength after dimension reduction, and these three characteristics are easy to obtain, convenient for model application and underground management.

**Table 3:** Comparison of results of multiple models.

Model	Parameter/model structure		Accuracy (%)		Time consumption/s
	*C*	*g*	Training set	Test set	
SVM	81.47	90.58	93.65 (59/63)	80.95 (17/21)	1.39
GWO-SVM	76.57	0.10	96.83 (61/63)	90.48 (19/21)	2.09
IGWO-SVM	84.32	0.97	100 (63/63)	100 (21/21)	2.21
RF-SVM	81.47	90.58	93.65 (59/63)	80.95 (17/21)	1.33
RF-GWO-SVM	61.21	0.06	96.83 (61/63)	90.48 (19/21)	2.49
RF-IGWO-SVM	68.67	0.42	100 (63/63)	100 (21/21)	2.61

## Conclusions

Through the theoretical research on the mechanism and process of coal and gas outburst, the predictive indexes of coal and gas outburst risk are reasonably determined, and the GWO and IGWO based on the Tent Chaotic Mapping and DLH strategy respectively are used to improve the predicting accuracy of the SVM. To solve the correlation and missing data among the data, the Random Forest algorithm is introduced to analyze the importance of multiple parameters, and the main prediction parameters are obtained to reconstruct the dataset for machine learning, and the following conclusions are drawn:

1. Following the mechanism of coal and gas outburst, eight quantitative parameters of outburst prediction can be summarized and sorted out, including the gas content, the gas pressure, the index of the initial velocity of diffusion of coal gas, the coefficient of firmness of coal, the distance from geological structure, the thickness of coal seams, the maximum drilling cuttings volume, and the initial velocity of gas emission from boreholes, and the relationship between each parameter and coal and gas outburst is explained.
2. The Grey Wolf Optimizer Algorithm (GWO) can effectively improve the prediction performance of the SVM model. GWO-SVM model has improved the accuracy of the test set to 90.48% from the original non-optimized 80.95%, which shows that the Grey Wolf optimization algorithm can better optimize the parameters ***C*** and ***g*** of SVM, and the introduction of this optimization algorithm can improve the performance of SVM greatly.
3. The prediction performance of SVM can be further improved by the Improved Grey Wolf Optimize Algorithm (IGWO). It can simplify the data and achieve the same prediction results after introducing the dimension reduction method of Random Forest. The IGWO is introduced to enhance the SVM model, and the prediction result is improved, reaching 100%, which proves the prediction performance of the IGWO-SVM model, and the optimized model can improve the original model's overfitting problem.
4. After the introduction of the Random Forest to reduce the dimension of the features, only three parameters with high importance are kept, which makes the data composition easier. When the data of the remaining features are missing, the model can still achieve better prediction performance. This can better meet the needs of predicting coal mine gas outburst areas and has certain practicability. This algorithm is based on an intelligent algorithm for risk identification, monitoring, and early warning of coal mine gas outburst disaster can provide a technical way for online monitoring, identifying, predicting, and early warning of hidden dangers of coal mine gas outburst disaster.

There are still some areas where our research needs to be improved. Due to the limitation of statistical ability, we can't guarantee that all the causes of coal and gas explosion risks are included. For example, some stress-related indicators such as energy amplitude and frequency, duration, and frequency band energy intensity obtained by micro-seismic monitoring technology, acoustic emission technology, and electromagnetic radiation technology are not included in the attribute list of this study. This may affect the results of risk prediction. In the future, the performance of the model will be further improved by adding more scientific dynamic parameter data to the research (Supplementary information).

### Supplementary Information

Supplementary Information.

## Data Availability

The data that support the findings of this study are available from the corresponding author upon reasonable request.
