# Supplementary material for: A novel combined intelligent algorithm prediction model for the risk of the coal and gas outburst
Source: Sci Rep. 2023 Sep 25;13:15988. doi: 10.1038/s41598-023-43013-0 (PMC10520021; doi:10.1038/s41598-023-43013-0)
Supplement: Supplementary file 1 — Supplementary Information. [file 41598_2023_43013_MOESM1_ESM.docx]

SVM Algorithm

tic

close all

clear

clc

format compact

Data= xlsread('data.xlsx');

data=Data(:,1:end-1);class=Data(:,end)+1;

data=knnimpute(data); % nan

m=4:4:length(class);

train_data = data; train_data(m,:)=[]; test_data = data(m,:);

train_labels = class; train_labels(m,:)=[]; test_labels = class(m,:);

%

% var = [2 1 ];

% var=[1 2 3 4 5 ];

% train_data = train_data(:,var);

% test_data = test_data(:,var);

[mtrain,ntrain] = size(train_data);

[mtest,ntest] = size(test_data);

dataset = [train_data;test_data];

[dataset_scale,ps] = mapminmax(dataset',0,1);

dataset_scale = dataset_scale';

train_data = dataset_scale(1:mtrain,:);

test_data = dataset_scale( (mtrain+1):(mtrain+mtest),: );

% tic;

% [bestCVaccuracy,bestc,bestg] = SVMcgForClass(train_labels,train_data)

% ga_option.maxgen = 100;

% ga_option.sizepop = 20;

% ga_option.ggap = 0.9;

% ga_option.cbound = [0,100];

% ga_option.gbound = [0,100];

% ga_option.v = 5;

% [bestacc,bestc,bestg] =gaSVMcgForClass(train_labels,train_data,ga_option)

% pso_option.c1 = 1.5;

% pso_option.c2 = 1.7;

% pso_option.maxgen = 100;

% pso_option.sizepop = 20;

% pso_option.k = 0.6;

% pso_option.wV = 1;

% pso_option.wP = 1;

% pso_option.v = 5;

% pso_option.popcmax = 100;

% pso_option.popcmin = 0.1;

% pso_option.popgmax = 100;

% pso_option.popgmin = 0.1;

% [bestacc,bestc,bestg] = psoSVMcgForClass(train_labels,train_data,pso_option)

% toc;

% cmd = ['-c ',num2str(bestc),' -g ',num2str(bestg)];

rng('default')

cmd = ['c ' num2str(rand(1)*100) ' -g ' num2str(rand(1)*100)]

% cmd = ['-c 100 -g 0.1']

model = svmtrain(train_labels, train_data,cmd);

[ptrain_label, train_accuracy, ~] = svmpredict(train_labels, train_data, model);

train_accuracy

[ptest_label, test_accuracy, ~] = svmpredict(test_labels, test_data, model);

test_accuracy

figure

plot(train_labels,'ro')

hold on;

plot(ptrain_label,'b*')

legend('Actual label', 'Predicting label')

xlabel(' Number of training sets ')

ylabel('Category')

title(' Training set prediction ')

ylim([0,3])

box off

figure

plot(test_labels,'ro')

hold on;

plot(ptest_label,'b*')

legend(' Actual label', 'Predicting label ')

xlabel(' Number of test sets '),ylabel('Category')

title(' Test set prediction '),ylim([0,3]),box off

toc

GWO-SVM Algorithm

tic %

%%

close all

clear

clc

format compact

%%

Data= xlsread('data.xlsx');

data=Data(:,1:end-1);class=Data(:,end)+1;

data=knnimpute(data); % nan

m=4:4:length(class);

train_data = data; train_data(m,:)=[]; test_data = data(m,:);

train_labels = class; train_labels(m,:)=[]; test_labels = class(m,:);

%

% var = [2 1 ];

% var=[1 2 3 ];

% train_data = train_data(:,var);

% test_data = test_data(:,var);

%%

%

[mtrain,ntrain] = size(train_data);

[mtest,ntest] = size(test_data);

dataset = [train_data;test_data];

[dataset_scale,ps] = mapminmax(dataset',0,1);

dataset_scale = dataset_scale';

train_data = dataset_scale(1:mtrain,:);

test_data = dataset_scale( (mtrain+1):(mtrain+mtest),: );

%%

SearchAgents_no=20;

Max_iteration=30;

dim=2;

lb=[0.01,0.01];

ub=[100,100];

v=10;

% initialize alpha, beta, and delta_pos

Alpha_pos=zeros(1,dim);

Alpha_score=inf;

Beta_pos=zeros(1,dim);

Beta_score=inf;

Delta_pos=zeros(1,dim);

Delta_score=inf;

%Initialize the positions of search agents

Positions=initialization(SearchAgents_no,dim,ub,lb);

Convergence_curve=zeros(1,Max_iteration);

train_accuracy=0;

% while train_accuracy<50

% while train_accuracy<97 | 100-bestGWOaccuarcy<93 | test_accuracy <90

SearchAgents_no=20;

Max_iteration=30;

dim=2; %

lb=[0.01,0.01]; %

ub=[100,100]; %

v=10;

% initialize alpha, beta, and delta_pos

Alpha_pos=zeros(1,dim);

Alpha_score=inf;

Beta_pos=zeros(1,dim);

Beta_score=inf;

Delta_pos=zeros(1,dim);

Delta_score=inf;

%Initialize the positions of search agents

Positions=initialization(SearchAgents_no,dim,ub,lb);

Convergence_curve=zeros(1,Max_iteration);

gen_num=0;

% Main loop

while gen_num<Max_iteration

for i=1:size(Positions,1)

Flag4ub=Positions(i,:)>ub;

Flag4lb=Positions(i,:)<lb;

Positions(i,:)=(Positions(i,:).*(~(Flag4ub+Flag4lb)))+ub.*Flag4ub+lb.*Flag4lb;

% cmd = [' -c ',num2str(Positions(i,1)),' -g ',num2str(Positions(i,2))];

% model=svmtrain(train_labels,train_data,cmd);

% [~,fitness(i)]=svmpredict(test_labels,test_data,model);

% [~,fitness(i)]=svmpredict(train_labels,train_data,model);

cmd_cv = ['-v ',num2str(v),' -c ',num2str(Positions(i,1)),' -g ',num2str(Positions(i,2))];

fitness(i) = svmtrain(train_labels,train_data,cmd_cv);

fitness(i)=100-fitness(i);

if fitness(i)<Alpha_score

Alpha_score=fitness(i);

Alpha_pos=Positions(i,:);

end

if fitness(i)>Alpha_score && fitness(i)<Beta_score

Beta_score=fitness(i);

Beta_pos=Positions(i,:);

end

if fitness(i)>Alpha_score && fitness(i)>Beta_score && fitness(i)<Delta_score

Delta_score=fitness(i);

Delta_pos=Positions(i,:);

end

end

a=2-gen_num*((2)/Max_iteration);

for i=1:size(Positions,1)

for j=1:size(Positions,2)

r1=rand(); % r1 is a random number in [0,1]

r2=rand(); % r2 is a random number in [0,1]

A1=2*a*r1-a;

C1=2*r2;

D_alpha=abs(C1*Alpha_pos(j)-Positions(i,j));

X1=Alpha_pos(j)-A1*D_alpha;

r1=rand();

r2=rand();

A2=2*a*r1-a;

C2=2*r2;

D_beta=abs(C2*Beta_pos(j)-Positions(i,j));

X2=Beta_pos(j)-A2*D_beta;

r1=rand();

r2=rand();

A3=2*a*r1-a;

C3=2*r2;

D_delta=abs(C3*Delta_pos(j)-Positions(i,j));

X3=Delta_pos(j)-A3*D_delta;

Positions(i,j)=(X1+X2+X3)/3;

end

end

gen_num=gen_num+1;

Convergence_curve(gen_num) = Alpha_score;

avgfitness_gen(gen_num,1) = mean(fitness);

end

bestc=Alpha_pos(1,1);

bestg=Alpha_pos(1,2);

% v=10;

% cmd_cv = ['-v ',num2str(v),' -c ',num2str(bestc),...

% ' -g ',num2str(bestg)];

% cv = svmtrain(train_labels,train_data,cmd_cv)

bestGWOaccuarcy=Alpha_score;

% end

toc

figure;

plot(100-Convergence_curve,'r*-','LineWidth',1.5);

hold on;plot(100-avgfitness_gen,'o-','LineWidth',1.5);

legend('The best fitting',' Average Fitting','Location','SouthEast');

xlabel('Iteration','FontSize',12);ylabel('Fitting','FontSize',12);

grid on;

line1 = ' Accuracy';

line2 = ['(c1=',num2str(bestc), ...

',c2=',num2str(bestg),',End iteration=', ...

num2str(Max_iteration),'pop=', ...

num2str(SearchAgents_no),')'];

title({line1;line2},'FontSize',12);

disp('Selecting result');

str=sprintf('Best Cross Validation Accuracy = %g%%，Best c = %g，Best g = %g',100-bestGWOaccuarcy,bestc,bestg);

disp(str)

cmd_gwosvm = ['-c ',num2str(bestc),' -g ',num2str(bestg)];

model_gwosvm = svmtrain(train_labels,train_data,cmd_gwosvm);

[ptrain_label, train_accuracy, ~] = svmpredict(train_labels, train_data, model_gwosvm);

train_accuracy

[ptest_label,test_accuracy, ~] = svmpredict(test_labels,test_data,model_gwosvm);

test_accuracy

total = length(test_labels);

right = sum(ptest_label == test_labels);

disp(' Print test set classification accuracy ');

str = sprintf( 'Accuracy = %g%% (%d/%d)',test_accuracy(1),right,total);

disp(str);

figure,plot(train_labels,'ro')

hold on;

plot(ptrain_label,'b*')

legend('Actual Label', 'Predicting Label')

xlabel(' Number of training sets ')

ylabel('Category')

title(' Training set prediction ')

ylim([0,3])

box off

figure,plot(test_labels,'ro'),hold on;

plot(ptest_label,'b*'),legend('Actual Label', 'Predicting Label')

xlabel(' Number of test sets '),ylabel('Category')

title(' Test set prediction '),ylim([0,3]),box off

toc

IGWO-SVM Algorithm

tic

close all

clear

clc

format compact

Data= xlsread('data.xlsx');

data=Data(:,1:end-1);class=Data(:,end)+1;

data=knnimpute(data); % nan

m=4:4:length(class);

train_data = data; train_data(m,:)=[]; test_data = data(m,:);

train_labels = class; train_labels(m,:)=[]; test_labels = class(m,:);

% var = [2 1];

var=[1 2 4 ];

% train_data = train_data(:,var);

% test_data = test_data(:,var);

[mtrain,ntrain] = size(train_data);

[mtest,ntest] = size(test_data);

dataset = [train_data;test_data];

[dataset_scale,ps] = mapminmax(dataset',0,1);%????????????????/

dataset_scale = dataset_scale';

train_data = dataset_scale(1:mtrain,:);

test_data = dataset_scale( (mtrain+1):(mtrain+mtest),: );

N=20;

Max_iter=30;

dim=2;

ub=[100 100];

lb=[0.01 0.01];

lu=[lb;ub];

trace=zeros(Max_iter,2);

%%

Alpha_pos=zeros(1,dim);

Alpha_score=inf; %change this to -inf for maximization problems

Beta_pos=zeros(1,dim);

Beta_score=inf; %change this to -inf for maximization problems

Delta_pos=zeros(1,dim);

Delta_score=inf; %change this to -inf for maximization problems

% Positions=initialization(N,dim,ub,lb);

X(1, :) = rand(1, dim);

for j = 1:dim

for i = 1:N-1

if X(i, j) < 0.6

X(i+1, j) = X(i, j)/0.6;

else

X(i+1, j) = (1-X(i, j))/0.4;

end

end

end

Positions = X.*(ub-lb)+lb;

% X(1, :) = rand(1, dim);

% for j = 1:dim

% for i = 1:N-1

% X(i+1, j) = 4*X(i, j)*(1-X(i, j));

% end

% end

% Positions = X.*(ub-lb)+lb;

% Positions = boundConstraint (Positions, Positions, lu);

% Calculate objective function for each wolf

for i=1:size(Positions,1)

Fit(i) =objfun(Positions(i,:),train_labels,train_data,test_labels,test_data);

end

% Personal best fitness and position obtained by each wolf

pBestScore = Fit;

pBest = Positions;

neighbor = zeros(N,N);

iter = 1;% Loop counter

%% Main loop

while iter <= Max_iter

for i=1:size(Positions,1)

fitness = Fit(i);

% Update Alpha, Beta, and Delta

if fitness<Alpha_score

Alpha_score=fitness; % Update alpha

Alpha_pos=Positions(i,:);

end

if fitness>Alpha_score && fitness<Beta_score

Beta_score=fitness; % Update beta

Beta_pos=Positions(i,:);

end

if fitness>Alpha_score && fitness>Beta_score && fitness<Delta_score

Delta_score=fitness; % Update delta

Delta_pos=Positions(i,:);

end

end

%% Calculate the candiadate position Xi-GWO

a=2-iter*((2)/Max_iter); % a decreases linearly from 2 to 0

% Update the Position of search agents including omegas

for i=1:size(Positions,1)

for j=1:size(Positions,2)

r1=rand(); % r1 is a random number in [0,1]

r2=rand(); % r2 is a random number in [0,1]

A1=2*a*r1-a; % Equation (3.3)

C1=2*r2; % Equation (3.4)

D_alpha=abs(C1*Alpha_pos(j)-Positions(i,j)); % Equation (3.5)-part 1

X1=Alpha_pos(j)-A1*D_alpha; % Equation (3.6)-part 1

r1=rand();

r2=rand();

A2=2*a*r1-a; % Equation (3.3)

C2=2*r2; % Equation (3.4)

D_beta=abs(C2*Beta_pos(j)-Positions(i,j)); % Equation (3.5)-part 2

X2=Beta_pos(j)-A2*D_beta; % Equation (3.6)-part 2

r1=rand();

r2=rand();

A3=2*a*r1-a; % Equation (3.3)

C3=2*r2; % Equation (3.4)

D_delta=abs(C3*Delta_pos(j)-Positions(i,j)); % Equation (3.5)-part 3

X3=Delta_pos(j)-A3*D_delta; % Equation (3.5)-part 3

X_GWO(i,j)=(X1+X2+X3)/3; % Equation (3.7)

end

X_GWO(i,:) = boundConstraint(X_GWO(i,:), Positions(i,:), lu);

Fit_GWO(i) =objfun(X_GWO(i,:),train_labels,train_data,test_labels,test_data);

end

%% Calculate the candiadate position Xi-DLH DLH改进的地方

radius = pdist2(Positions, X_GWO, 'euclidean'); % Equation (10)

dist_Position = squareform(pdist(Positions));

r1 = randperm(N,N);

for t=1:N

neighbor(t,:) = (dist_Position(t,:)<=radius(t,t));

[~,Idx] = find(neighbor(t,:)==1); % Equation (11)

random_Idx_neighbor = randi(size(Idx,2),1,dim);

for d=1:dim

X_DLH(t,d) = Positions(t,d) + rand .*(Positions(Idx(random_Idx_neighbor(d)),d)...

- Positions(r1(t),d)); % Equation (12)

end

X_DLH(t,:) = boundConstraint(X_DLH(t,:), Positions(t,:), lu);

Fit_DLH(t) = objfun(X_DLH(t,:),train_labels,train_data,test_labels,test_data);

end

%% Selection

tmp = Fit_GWO < Fit_DLH; % Equation (13)

tmp_rep = repmat(tmp',1,dim);

tmpFit = tmp .* Fit_GWO + (1-tmp) .* Fit_DLH;

tmpPositions = tmp_rep .* X_GWO + (1-tmp_rep) .* X_DLH;

%% Updating

tmp = pBestScore <= tmpFit; % Equation (13)

tmp_rep = repmat(tmp',1,dim);

pBestScore = tmp .* pBestScore + (1-tmp) .* tmpFit;

pBest = tmp_rep .* pBest + (1-tmp_rep) .* tmpPositions;

Fit = pBestScore;

Positions = pBest;

[~,index]=sort(Fit);

trace(iter,1)=1-Fit(index(1)) ;

trace(iter,2)=1-sum(Fit)./length(Fit);

GlobalParams=Positions(index(1),:);

%%

iter = iter+1;

neighbor = zeros(N,N);

end

bestc=GlobalParams(1);

bestg=GlobalParams(2);

figure;

hold on;

trace = round(trace*10000)/10000;

plot(trace(1:Max_iter,1),'r*-','LineWidth',1.5);

plot(trace(1:Max_iter,2),'o-','LineWidth',1.5);

legend('The best fitting', 'Average fitting');

xlabel('iteration','FontSize',12);

ylabel('fitting','FontSize',12);

axis([0 Max_iter-1 0 100]);

grid on;

axis auto;

line1 = ' Accuracy[DIH-GWOmethod]';

line2 = ['(End iteration=', ...

num2str(Max_iter),', pop=', ...

num2str(N),')'];

line3 = ['Best c=',num2str(bestc),' g=',num2str(bestg), ...

' CVAccuracy=',num2str((1-min(Fit))*100),'%'];

title({line1;line2;line3},'FontSize',12);

disp(' Print selection results ');

str=sprintf('Best c = %g，Best g = %g',bestc,bestg);

disp(str)

cmd_gwosvm = ['-c ',num2str(bestc),' -g ',num2str(bestg)];

model_gwosvm = svmtrain(train_labels,train_data,cmd_gwosvm);

[ptrain_label, train_accuracy, ~] = svmpredict(train_labels, train_data, model_gwosvm);

train_accuracy

[ptest_label,test_accuracy, ~] = svmpredict(test_labels,test_data,model_gwosvm);

test_accuracy

total = length(test_labels);

right = sum(ptest_label == test_labels);

disp(' Print test set classification accuracy ');

str = sprintf( 'Accuracy = %g%% (%d/%d)',test_accuracy(1),right,total);

disp(str);

figure,plot(train_labels,'ro')

hold on;

plot(ptrain_label,'b*')

legend('Actual label', 'Predicting label')

xlabel(' Number of training sets ')

ylabel('Category')

title(' Training set prediction ')

ylim([0,3])

box off

figure,plot(test_labels,'ro'),hold on;

plot(ptest_label,'b*'),legend('Actual label', 'Predicting label')

xlabel(' Number of test sets '),ylabel('Category')

title(' Test set prediction '),ylim([0,3]),box off

toc

Random Forest Algorithm

% train_data = x1;

% train_labels = y1;

leaf=5;

ntrees=200;

fboot=1;

surrogate='on';

disp('Training the tree bagger')

b = TreeBagger(...

ntrees,...

train_data,train_labels,...

'Method','classification',...

'oobvarimp','on',...

'surrogate',surrogate,...

'minleaf',leaf,...

'FBoot',fboot);

% 'FBoot',fboot,...

% 'Options',paroptions...

% );

% plot(x,x,'LineWidth',3);

%

% hold on

% scatter(x,y,'filled');

% hold off

% grid on

%

% set(gca,'FontSize',18)

% xlabel('Actual','FontSize',25)

% ylabel('Estimated','FontSize',25)

% title(['Training Dataset, R^2=' num2str(cct^2,2)],'FontSize',30)

drawnow

% Calculate the relative importance of the input variables

disp('Sorting importance into descending order')

weights=b.OOBPermutedVarDeltaError;

[B,iranked] = sort(weights,'descend');

%--------------------------------------------------------------------------

disp(['Plotting a horizontal bar graph of sorted labeled weights.'])

%--------------------------------------------------------------------------

figure

barh(weights(iranked(1:8)),'g');

xlabel('Variable Importance','FontSize',30,'Interpreter','latex');

ylabel('Variable Rank','FontSize',30,'Interpreter','latex');

title(...

['Relative Importance of Inputs in estimating Redshift'],...

'FontSize',17,'Interpreter','latex'...

);

hold on

barh(weights(iranked(1:5)),'y');

barh(weights(iranked(1:3)),'r');

set(gca,'yticklabel',{'x_1_','x_2_','x_3_','x_4_','x_5_','x_6_','x_7_','x_8_'})
